# Cognitive decline before and after mid-to-late-life continuing education in England: a matched longitudinal analysis of a prospective cohort study

**DOI:** 10.1016/j.lanepe.2025.101513

**Published:** 2025-10-29

**Authors:** Mikaela Bloomberg, Séverine Sabia, Feifei Bu, Jessica Gong, Andrew Steptoe

**Affiliations:** a Department of Epidemiology and Public Health, University College London, London, UK; b Université Paris Cité, Inserm U1153, Center for Research in Epidemiology and Statistics (CRESS), Epidemiology of Ageing and Neurodegenerative diseases (EpiAgeing), Paris, France; c Faculty of Brain Sciences, University College London, London, UK; d Department of Behavioural Science and Health, University College London, London, UK

**Keywords:** Cognitive ageing, Cognitive function, Continuing education, Adult education, Cognitive decline

## Abstract

**Background:**

While WHO/Europe guidelines promote life-long learning for healthy ageing generally, whether adult education supports healthy cognitive ageing is unclear. We used a matched longitudinal study design to account for reverse causality seen in previous studies and assess whether cognitive trajectories improved following mid-to-late life education using data from a nationally representative UK-based cohort study.

**Methods:**

Data were drawn from 3906 participants aged 50–90 years followed up from 2002-03 to 2021-23. At biennial interviews, participants were asked to report whether they had engaged in continuing education in the last 12 months and were split into ‘single’ (continuing education at one interview) and ‘multiple’ (continuing education at multiple interviews) continuing education groups. We used piecewise linear mixed models to examine memory and fluency decline four years before and eight years after first report of continuing education (corresponding to the median follow-up period) and during the comparable period in a matched control group.

**Findings:**

Cognitive trajectories were comparable between continuing education groups and their controls before participation in continuing education. After participation, cognitive trajectories were still similar; e.g., the difference in eight-year memory decline between the single continuing education group and controls was just −0.026 standard deviations (95% CI = −0.081–0.029) or −0.015 standard deviations (95% CI = −0.067–0.037) between the multiple continuing education group and controls.

**Interpretation:**

There was no evidence of improvement in cognitive trajectories following continuing education. These findings suggest continuing education should not yet be prioritised as a strategy for long-term cognitive health until further evidence demonstrates a clear benefit.

**Funding:**

10.13039/501100000272National Institute for Health and Care Research, 10.13039/100000049National Institute on Aging.

**Research in context**

**Evidence before this study**

We searched PubMed for publications until 1 July 2025 using search terms “adult education”, “continuing education”, “cognitive performance”, “cognitive function”, “cognitive decline,” and “cognitive ageing”. While there is robust evidence linking early life education and late-life cognitive health, several observational studies have reported that continuing education undertaken in mid-to-late life is also associated with better cognitive performance and a lower risk of dementia. Lifelong learning is increasingly promoted in policy discourse across Europe as part of healthy ageing strategies, with the expectation that it supports social participation, wellbeing, and active engagement in later life.

**Added value of this study**

Although continuing education is relevant for other healthy ageing outcomes, its impact on long-term cognitive health remains uncertain. Most existing studies are observational and prone to reverse causality—that is, individuals with better cognitive health may be more likely to pursue continuing education. In this study, we used a matched longitudinal study design with quasi-experimental elements, including comparison of cognitive trajectories before and after education with a well-matched control group. Drawing on 21 years of data from a largescale, nationally representative English cohort study, we found no evidence that continuing education alters longer-term cognitive trajectories. This suggests previous results largely reflect shorter-term cognitive improvement or reverse causality.

**Implications of all the available evidence**

While continuing education has important social, psychological, and wellbeing benefits, and may provide short-term cognitive engagement, its role in promoting long-term cognitive health may be limited. By contrast, there is consistent and robust evidence supporting the importance of early-life education for lifelong cognitive function. Policy aiming to promote healthy cognitive ageing should therefore continue to prioritise equitable access to high-quality education in childhood and early adulthood, while recognising that later-life education requires further evidence before being positioned as a strategy for long-term cognitive health.

## Introduction

Alzheimer's disease (AD) and related dementias represent a pressing threat to public health in Europe, with an estimated prevalence of 12.7 million cases in 2019.^1^ As the population ages, the number of people living with dementia is projected to increase, with a forecasted European prevalence of 22.8 million cases by 2050.^1^ Diagnosis of AD—the most prevalent cause of dementia—typically follows a prolonged period of neuropathological changes and cognitive decline.^2^ Given this extended preclinical phase and a paucity of effective disease-modifying treatments, research efforts have focussed on identifying modifiable risk factors for cognitive decline from early life onward.^3^

Of these factors, education is among the most commonly researched, where higher early-life educational attainment is consistently associated with improvement across cognitive outcomes.^3^ There is also some evidence to suggest that education undertaken during mid-to-late life is associated with better cognitive performance and reduced dementia risk.^4^, ^5^, ^6^, ^7^, ^8^, ^9^, ^10^ Nevertheless, this body of literature has a key methodological limitation: individuals with poor cognitive function or accelerated cognitive decline are less likely to participate in adult education. As a result, it is not possible to determine whether better cognitive health predisposes older adults to pursue continuing education, or whether continuing education leads to an improvement in cognitive health.

As dementia prevention efforts across Europe increasingly emphasise modifiable risk factors in line with World Health Organization (WHO) guidelines,^11^ it is essential to distinguish between activities that offer short-term cognitive engagement or broader wellbeing benefits and those that meaningfully alter long-term cognitive ageing to design effective and equitable policy. In the present study, we made use of 21 years of cognitive data from 3906 participants aged 50–90 years in the English Longitudinal Study of Ageing (ELSA). To determine whether cognitive ageing trajectories improved following mid-to-late life continuing education, we compared cognitive decline before and after engaging in continuing education to a group of matched controls during the comparable time period. This quasi-experimental design allowed us to address the question of reverse causality seen in previous studies.

## Methods

### Data sources

ELSA is a nationally representative cohort study of community-dwelling adults aged ≥50 years living in England. Data collection began in 2002–03 (wave 1) and was repeated biennially thereafter until 2018–19 (wave 9); wave 10 took place in 2021–23. Further details of survey design and implementation are available elsewhere.^12^ Each wave of ELSA receives the relevant research ethics approval; the most recent (22 May 2023) was from the South Central—Berkshire Research Ethics Committee (23/SC/0112). Written informed consent was obtained at each interview. No further ethical approval was required for this secondary analysis.

Data from waves 1 to 10 of ELSA were used in the present study. ELSA respondents aged ≥50 who did not report continuing education at baseline and had at least two waves of data collection were eligible for inclusion in the analysis. For this study, the ‘baseline’ for each participant was considered to be the first wave with complete cognitive and covariate data. We excluded participants who reported continuing education at their baseline wave, as they lacked a pre-continuing education cognitive assessment within the study period. Several participants (N = 93) reported continuing education at waves prior to their analytic baseline. Of the 93, 14 did not report continuing education again during the ELSA study period, while the remainder subsequently reported continuing education. Given the relatively small number and the inherent uncertainty around timing and duration of continuing education prior to study enrolment, these individuals were retained.

### Continuing education

At each interview, participants were asked if they had “attended a formal educational or training course” in the last month or last 12 months. Participants who answered yes to either of these questions were categorised as the continuing education group. The continuing education group was further subdivided into ‘single continuing education’ (reporting continuing education at a single wave) and ‘multiple continuing education’ (reporting continuing education at multiple waves).

### Cognitive assessment

The cognitive domains examined were episodic memory and verbal fluency, which are key for day-to-day function and show decline with dementia.^13^ Memory was assessed using immediate and delayed recall tasks.^14^ In each of these tasks, participants were read a 10-word list and asked to recall it immediately (range: 0–10) and after a short delay (range: 0–10). These scores were summed to give a summary recall score (range: 0–20). At each wave, participants were given a different list of words to memorise to reduce practice effects. Fluency was assessed using the animal naming task,^15^ where participants were asked to name out loud as many animals as possible in 1-min. Memory was assessed at every wave and fluency at all waves except wave 6. Cognitive test scores were standardised across the full ELSA study population over waves 1–10, such that each domain had a mean of 0 and standard deviation of 1 in the pooled sample.

### Other covariates

Covariates were selected on the basis of associations with continuing education and cognitive performance and were self-reported. These included birth year, sex (male or female; ELSA survey materials refer to sex and not gender), baseline education level (less than high school, high school, or above high school), labour force status (employed, unemployed/disabled, retired, homemaker/other), household non-pension wealth (standardised by year), smoking status (current smoker or not), alcohol consumption (consumes alcohol or not), physical activity level (engages in moderate-vigorous physical activity [MVPA] weekly or not), self-rated hearing (excellent, very good, good, fair, or poor), depressive symptoms (based on the 8-item Center for Epidemiologic Studies Depression [CES-D] scale; Methods S1), and self-report of clinical diagnosis of the following long-term conditions (yes or no): high blood pressure, heart disease, stroke, cancer, diabetes, and psychiatric conditions.

### Matching

In similar methods to previous studies,^16^^,^^17^ to establish whether cognitive trajectories changed following continuing education, we selected a matched control group of comparable participants who reported no continuing education during the analytic period (‘controls’). We used coarsened exact matching, a highly flexible matching method that is robust to measurement error, requires fewer assumptions than other methods, and does not require the iterative checking process to guarantee balance necessary for other methods.^18^

In coarsened exact matching, controls are assigned to a matching stratum with individuals in the continuing education group of similar characteristics, where the researcher specifies the degree of similarity for each matching characteristic. To allow for causal inference without the use of model weights, we used the k-to-k procedure in which controls are randomly dropped from each stratum to equalise the number of continuing education and control participants in each matching stratum. In the present analysis, matching criteria were baseline age, sex, baseline education level, baseline mean standardised cognitive performance (i.e., the mean of standardised memory and fluency performance), and follow-up duration. As each additional matching variable leads to a further reduction in sample size because more participants fail to find matches, these matching variables were chosen as the most essential for comparability of the exposure groups; other confounders were accounted for by model adjustment. Matching on age and cognitive score at baseline gave us comparison groups with similar cognitive performance at a similar age, enhancing the cognitive comparability of the two groups. Matching on age at baseline also equivalises selection bias due to survival between groups.

Because we matched on baseline cognitive performance, we did not exclude participants with dementia or cognitive impairment to include the full range of cognitive health in each group. Matching on cognitive performance also helped mitigate potential impacts of prior continuing education on the results, as some individuals may have already participated in continuing education before the study period. Further details of matching are available in the Supplementary Materials (Methods S2).

To be able to examine people who repeatedly engaged in continuing education separately from those who reported just one instance of continuing education, we selected two groups of controls. One group of controls was matched to the multiple continuing education group and the other group of controls was matched to the single continuing education group. Participants who reported no continuing education could be in either or both control groups.

### Statistical analysis

We used piecewise linear mixed models to examine differences in memory and fluency trajectories between continuing education and control groups, with separate models for each cognitive domain. Linear mixed models use all available data, and robustly handle non-monotone missingness patterns and item missingness, including attrition, assuming data missing-at-random.^19^ Baseline covariates with missing values were handled using complete-case analysis, given the low proportion of missingness and the complexity of integrating multiple imputation with the matching procedure.

All models included a random intercept and two random slopes (one for before the first instance of continuing education and one for after) at the individual level (with an unstructured covariance matrix) to account for interindividual differences in cognitive trajectories. Standard errors were derived from the mixed model covariance structure; additional clustering by matched set was not applied. Analyses were performed separately for: 1) single continuing education and their controls; and 2) multiple continuing education and their controls.

We modelled cognitive trajectories using piecewise polynomial functions with a knot at time = 0 ($t_{0}$) representing the age ($age_{t0}$) at which each participant in the continuing education group first reported engaging in continuing education. Each participant's follow-up period was centred at $t_{0}$ to capture changes in cognitive trajectory before and after continuing education. To ensure that the continuing education and control groups were compared over a similar age range, we aligned the control group's time scale accordingly. Within each matching stratum, we calculated the median $age_{t0}$ for the continuing education group. Then, for the control group in that same stratum, we assigned time=0 at that median age.

Two separate time terms were included in the models to correspond to the periods before and from t0: ‘pre-time’ for t<0 and ‘post-time’ for t≥0. Where interactions are denoted using ‘x’, all models included education group (continuing education or control), pre-time, post-time, education group x pre-time, and education group x post-time. All models also included $age_{t0}$, $age_{t0}$ x pre-time, and $age_{t0}$ x post-time, to account for remaining differences in $age_{t0}$ between education groups and to allow for cognitive trajectories to differ depending on $age_{t0}$. Using backward selection, we examined whether to include higher order time terms (quadratic and cubic) and interactions of these terms with education group or $age_{t0}$ to fully account for nonlinearity of cognitive trajectories. These terms were retained where significant (p < 0.05) on the basis of the Wald test, leading us to include post-time^2^ in memory models for both the single and multiple continuing education groups, and post-time^2^ in fluency models for the multiple continuing education group only. We assessed the suitability of these functional forms by fitting cubic spline models of memory and fluency trajectories over the full observed range of follow-up, with knots at the 5th, 35th, 65th, and 95th percentiles of follow-up time (Figures S1 and S2).

Finally, to account for remaining imbalance between the continuing education and control groups before $t_{0}$ due to variable coarsening and other confounders, all models were adjusted for birth year, sex, education level at baseline, labour force status, wealth, health behaviours, self-rated hearing, depressive symptoms, and long-term conditions. To fully account for differences between the continuing education group and controls in the period before continuing education whilst not adjusting for mediators following continuing education, time-updating variables (labour force status, wealth, smoking status, alcohol consumption, physical activity level, self-rated hearing, depressive symptoms, and long-term conditions) were allowed to vary with time until $age_{t0}$ and then carried forward (i.e., fixed) for the post-continuing-education period.

We used postestimation predictions from the fitted mixed models (Methods S3) to derive marginal estimates for cognitive decline in the continuing education and control groups and the difference in cognitive decline between groups in the four years before $t_{0}$ and eight years after $t_{0}$ (corresponding to the median follow-up periods before and after $t_{0}$). Finally, we plotted predicted marginal cognitive trajectories during these time periods. All analyses were performed in StataMP 18.0 with a two-sided p < 0.05 considered statistically significant.

### Additional analyses

We first examined variation in results by $age_{t0}$, by sex, and by education level (Methods S4). We next assessed the role of practice effect by rerunning the analyses including an indicator for assessment round.

To determine whether there was a discrete improvement in cognitive performance at $t_{0}$ that could have influenced pre $t_{0}$ slope due to constraining the model intercepts to be the same before and at $t_{0}$, we reran analyses including additional terms in the model (a discontinuity indicator evaluating to 1 if t≥0 and 0 otherwise and interactions between this discontinuity indicator and education group) to allow for a step-change in cognitive performance at $t_{0}$.

Finally, we ran a series of sensitivity analyses in subsets of the analytic sample to explore the robustness of the results. As these analyses required re-matching a subset of participants, we matched and analysed the single and multiple continuing education groups together to avoid small sample sizes. First, to isolate the impact of more structured and sustained educational experiences, we restricted the continuing education group to those who reported gaining an additional qualification (Methods S4). Next, we reran analyses separately in participants with and without evidence of cognitive impairment or dementia (Methods S4) to determine whether results differed by cognitive status. Finally, to clarify exposure timing, we restricted the continuing education group to those reporting continuing education in the past month only.

### Role of the funding source

Study sponsors were not involved in study design, the collection, analysis or interpretation of data, writing of the report, or the decision to submit the paper for publication.

## Results

### Participant characteristics

**Fig. 1:**
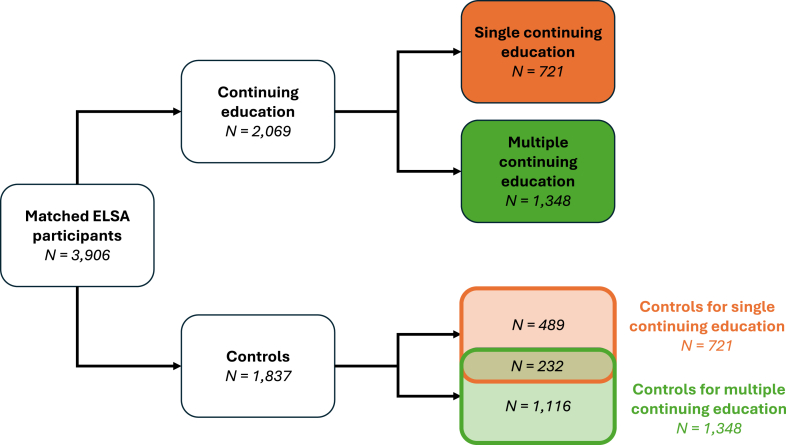
**Sample description.** Controls refer to individuals who did not report continuing education during the follow-up period. Single continuing education group refers to those reporting continuing education at a single wave; multiple continuing education refers to individuals reporting continuing education at multiple waves. The single and multiple continuing education groups are analysed separately. Each analysis (single or multiple continuing education) includes an equal number in the continuing education and control groups, with some overlap (N = 232) in the control group between the two analyses.

Of 21,192 ELSA respondents aged ≥50, 2809 (13.3%) did not participate in cognitive testing, 603 (2.8%) were missing covariates, 2582 (12.2%) reported continuing education at their baseline interview, and 3120 (14.7%) had <2 waves of data. Of the remaining 12,078 participants, 2291 (19.0%) reported continuing education. Of these 2291 participants, 2069 (90.3%; 721 in the single and 1348 in the multiple continuing education group) were matched with controls to give a final analytic sample of 3906 participants (Figure S3). Some controls (N = 232) were matched to both continuing education groups, resulting in a total number of controls (N = 1837) that is smaller than the number of continuing education participants (Fig. 1). ELSA participants excluded from the analytic sample were older, less educated, less wealthy, less healthy, and had lower cognitive scores than included participants (Table S1). Participants in continuing education groups excluded during matching were generally similar to included continuing education participants, but were slightly older, more educated, more likely to be retired, wealthier, and had higher cognitive scores (Table S2).

Participant characteristics at baseline are presented in Table 1 (single continuing education) and Table 2 (multiple continuing education). At baseline, the continuing education groups and their respective controls were similar in age (mean overall = 57.6 years, standard deviation [SD] = 7.2), birth year, sex (55.4% female overall), education level at baseline (54.5% educated to high school level and 21.0% educated above high school level overall), wealth, self-rated hearing, prevalence of most chronic conditions, and baseline cognitive performance. The single continuing education group was less likely than controls to smoke. The multiple continuing education group was more likely than controls to consume alcohol, had fewer depressive symptoms, and were less likely to report diabetes and psychiatric conditions. Both continuing education groups were more likely than their respective controls to be employed and to report weekly MVPA.

**Table 1:** Participants characteristics at baseline (single continuing education and controls).

	Control N = 721	Continuing education N = 721	p-value
Age in years, mean (SD)	56.7 (6.2)	56.5 (6.3)	0.51
Birth year, mean (SD)	1947.8 (8.1)	1947.9 (8.0)	0.79
Sex			
Male	322 (44.7)	322 (44.7)	
Female	399 (55.3)	399 (55.3)	>0.99
Education level			
Below high school	140 (19.4)	140 (19.4)	
High school	384 (53.3)	384 (53.3)	>0.99
Above high school	197 (27.3)	197 (27.3)	
Labour force status			
Employed	420 (58.3)	514 (71.3)	
Retired	179 (24.8)	135 (18.7)	<0.0001
Unemployed/disabled	62 (8.6)	32 (4.4)	
Homemaker/other	60 (8.3)	40 (5.5)	
Standardised wealth, mean (SD)	0.20 (1.4)	0.22 (1.3)	0.79
Current smoker	136 (18.9)	95 (13.2)	0.0041
Currently consumes alcohol	673 (93.3)	670 (92.9)	0.84
Reports weekly MVPA	602 (83.5)	638 (88.5)	0.0079
Self-rated hearing			
Excellent	192 (26.6)	210 (29.1)	
Very good	226 (31.3)	200 (27.7)	0.085
Good	191 (26.5)	224 (31.1)	
Fair	97 (13.5)	76 (10.5)	
Poor	15 (2.1)	11 (1.5)	
CES-D score, mean (SD)	1.3 (1.9)	1.3 (1.8)	0.57
Diagnosis of:			
High blood pressure	186 (25.8)	177 (24.5)	0.63
Heart disease	4 (0.6)	8 (1.1)	0.38
Stroke	67 (9.3)	48 (6.7)	0.080
Cancer	29 (4.0)	45 (6.2)	0.073
Diabetes	31 (4.3)	27 (3.7)	0.69
Psychiatric conditions	68 (9.4)	60 (8.3)	0.52
Cognitive scores, mean (SD)			
Standardised memory	0.35 (0.77)	0.34 (0.77)	0.97
Standardised fluency	0.28 (0.86)	0.29 (0.81)	0.81
Standardised mean	0.36 (0.71)	0.36 (0.70)	0.85

N (%) shown unless otherwise indicated.

Abbreviations: SD, standard deviation; MVPA, moderate-to-vigorous physical activity; CES-D, Center for Epidemiologic Studies Depression.

**Table 2:** Participants characteristics at baseline (multiple continuing education and controls).

	Control N = 1348	Continuing education N = 1348	p-value
Age in years, mean (SD)	58.1 (7.5)	57.9 (7.5)	0.56
Birth year, mean (SD)	1947.0 (10.0)	1947.2 (10.0)	0.75
Sex			
Male	600 (44.5)	600 (44.5)	
Female	748 (55.5)	748 (55.5)	>0.99
Education level			
Below high school	350 (26.0)	350 (26.0)	
High school	743 (55.1)	743 (55.1)	>0.99
Above high school	255 (18.9)	255 (18.9)	
Labour force status			
Employed	683 (50.7)	846 (62.8)	
Retired	390 (28.9)	337 (25.0)	<0.0001
Unemployed/disabled	127 (9.4)	75 (5.6)	
Homemaker/other	148 (11.0)	90 (6.7)	
Standardised wealth, mean (SD)	0.05 (1.1)	0.08 (1.0)	0.51
Current smoker	244 (18.1)	223 (16.5)	0.31
Currently consumes alcohol	1226 (90.9)	1265 (93.8)	0.0058
Reports weekly MVPA	1093 (81.1)	1169 (86.7)	<0.0001
Self-rated hearing			
Excellent	348 (25.8)	379 (28.1)	
Very good	387 (28.7)	378 (28.0)	0.71
Good	399 (29.6)	393 (29.2)	
Fair	181 (13.4)	166 (12.3)	
Poor	33 (2.4)	32 (2.4)	
CES-D score, mean (SD)	1.5 (2.0)	1.3 (1.8)	0.0076
Diagnosis of:			
High blood pressure	379 (28.1)	382 (28.3)	0.93
Heart disease	21 (1.6)	12 (0.9)	0.16
Stroke	152 (11.3)	149 (11.1)	0.90
Cancer	61 (4.5)	74 (5.5)	0.29
Diabetes	72 (5.3)	47 (3.5)	0.024
Psychiatric conditions	134 (9.9)	95 (7.0)	0.0087
Cognitive scores, mean (SD)			
Standardised memory	0.21 (0.83)	0.22 (0.84)	0.61
Standardised fluency	0.14 (0.84)	0.15 (0.83)	0.77
Standardised mean	0.20 (0.78)	0.22 (0.77)	0.61

N (%) shown unless otherwise indicated.

Standardised mean cognitive score corresponds to mean of standardised memory and fluency scores.

Abbreviations: SD, standard deviation; MVPA, moderate-to-vigorous physical activity; CES-D, Center for Epidemiologic Studies Depression.

The $age_{t0}$ in continuing education groups was similar to controls (mean 62.2 years [SD = 7.6] compared with 62.0 years [SD = 7.8] for controls). The absolute difference in $age_{t0}$ between the continuing education groups and controls in their matching stratum was ≤0.5 years for 75% of participants, ≤3.0 years for 90% of participants, and ≤5.0 years for 95% of participants. Follow-up periods were also similar: before $t_{0}$, the median follow-up was 3.0 years (interquartile range [IQR] = 2.0–6.0) for continuing education groups and 4.0 years (IQR = 2.0–6.0) for control groups; after $t_{0}$, the median follow-up was 8.0 years (IQR = 4.0–13.0) for both the continuing education and control groups. Attrition rates by wave were similar in continuing education and control groups (Tables S3 and S4).

### Continuing education and cognitive decline

#### Single continuing education

**Fig. 2:**
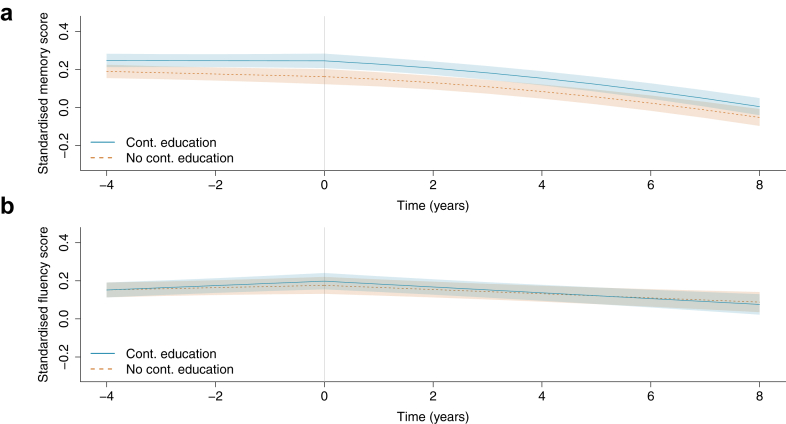
**Average cognitive trajectories before and after continuing education in the single continuing education group and controls (N = 1442).** Predicted cognitive scores averaged over the observed covariate distribution in the analytic sample, shown over the median follow-up periods of four years before and eight years after continuing education (time = 0) or the equivalent time interval in the control group. Panel A shows memory and Panel B fluency. The continuing education group includes 721 participants who reported just one wave of continuing education during the follow-up period. Models were adjusted for age at time = 0, sex, baseline education level, labour force status, wealth, health behaviours, self-rated hearing, depressive symptoms, and long-term conditions.

Memory was stable in the four years prior to continuing education in both the single continuing education group and controls (Fig. 2, panel A), with an average change of −0.002 SD (95% confidence interval [CI] = −0.029 to 0.025) in the single continuing education group and −0.027 SD (−0.057 to 0.002) in controls (difference = 0.025, 95% CI = −0.010 to 0.061, p = 0.16). In the eight years following continuing education, memory declined in both groups at a similar rate, with an average change of −0.240 SD (−0.283 to −0.198) in the continuing education group and −0.214 SD (−0.258 to −0.171) in controls (difference = −0.026 SD, 95% CI = −0.081 to 0.029; p = 0.35). The covariance between the random intercept and post-t_0_ slope was −0.002 (−0.005 to 0.000), with a corresponding correlation coefficient (ρ) of −0.11. The minimum detectable effect size (MDES) for post-$t_{0}$ slope difference between the continuing education and control groups was 0.078 SD over eight years.

In the four years prior to continuing education, fluency scores were relatively stable or even improved slightly in both the single continuing education group and controls (Fig. 2, panel B). The average change in the continuing education group was 0.046 SD (0.017–0.075) or 0.024 SD (−0.007 to 0.055) in controls (difference = 0.022, 95% CI = −0.016 to 0.061, p = 0.25). Fluency declined at a similar rate in both groups in the eight years after continuing education, with an average change of −0.122 SD (−0.172 to −0.072) for the continuing education group and −0.088 SD (−0.138 to −0.037) for controls (difference = −0.034 SD, 95% CI = −0.105 to 0.037, p = 0.34). The random intercept and post-t_0_ slope covariance was −0.006 (−0.010 to −0.003; ρ = −0.19). The MDES for the post-$t_{0}$ slope difference was 0.101 SD over eight years.

#### Multiple continuing education

**Fig. 3:**
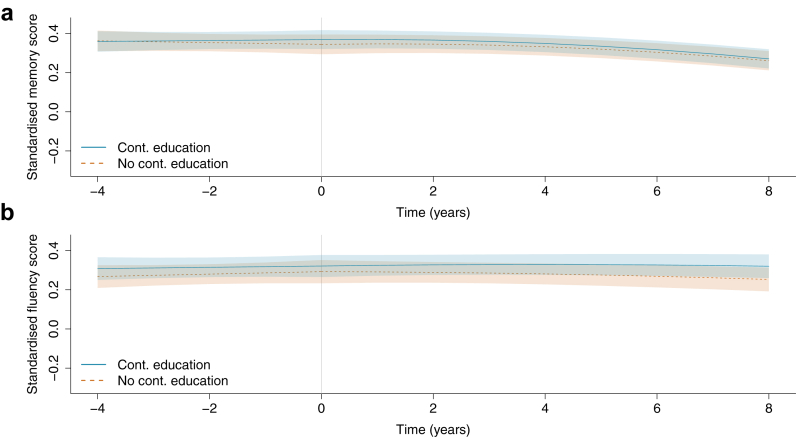
**Average cognitive trajectories before and after continuing education in the multiple continuing education group and controls (N = 2696).** Predicted cognitive scores averaged over the observed covariate distribution in the analytic sample, shown over the median follow-up periods of four years before and eight years after continuing education (time = 0) or the equivalent time interval in the control group. Panel A shows memory and Panel B fluency. The continuing education group includes 1348 participants who reported multiple waves of continuing education during the follow-up period. Models were adjusted for age at time = 0, sex, baseline education level, labour force status, wealth, health behaviours, self-rated hearing, depressive symptoms, and long-term conditions.

Memory was stable in the four years prior to continuing education in both the multiple continuing education and control groups (Fig. 3, panel A), with an average change of 0.010 SD (−0.040 to 0.060) in the continuing education group and −0.018 SD (−0.071 to 0.035) for controls (difference = 0.028, 95% CI = −0.038 to 0.093, p = 0.41). In the eight years following the first instance of continuing education, memory declined at a similar rate in both groups, with an average change of −0.099 SD (−0.142 to −0.055) in the continuing education group and −0.084 SD (−0.130 to −0.038) in controls (difference = −0.015, 95% CI = −0.067 to 0.037, p = 0.57). The random intercept and post-t_0_ slope covariance was 0.000 (−0.002 to 0.003; ρ = 0.03). The MDES for the post-$t_{0}$ slope difference was 0.074 SD over eight years.

In the four years prior to continuing education, fluency scores were stable in both the multiple continuing education and control groups (Fig. 3, panel B). The average change in the continuing education group was 0.013 SD (−0.042 to 0.069) or 0.025 SD (−0.035 to 0.084) for controls (difference = −0.012, 95% CI = −0.085 to 0.061, p = 0.75). Fluency scores were also relatively stable in the eight years after first instance of continuing education, with an average change of −0.001 SD (−0.058 to 0.056) for the continuing education group and −0.040 SD (−0.100 to 0.021) for controls (difference = 0.039, 95% CI = −0.034 to 0.112, p = 0.30). The random intercept and post-t_0_ slope covariance was −0.003 (−0.007 to 0.001; ρ = −0.11). The MDES for the post-$t_{0}$ slope difference was 0.103 SD over eight years.

### Additional analysis

Findings were consistent with no cognitive benefit across sex, age, and education levels (Tables S5–S7) and after adjustment for practice effect (Figures S4 and S5).

Stepwise changes in cognitive performance at $t_{0}$ were generally small. Controls showed minor reductions in cognitive performance, while continuing education groups showed minor increases; these stepwise changes were not statistically significant in most cases (Table S8). The exception was memory in the single continuing education group, where the stepwise increase was larger than in controls (p = 0.012), leading to a difference in memory performance of 0.111 SD (0.053–0.169) at $t_{0}$. However, this difference was attenuated over follow-up; by 8 years after $t_{0}$, the difference in scores between groups was similar (0.058, 95% CI = −0.004 to 0.119; p = 0.066).

There was also no evidence of improvement in cognitive decline following continuing education when the continuing education group included only those gaining educational qualifications (N = 1102 participants total; Figure S6), when analyses were restricted to those with (N = 920) or without (N = 1790) evidence of cognitive impairment or dementia (as defined in Methods S3; Figures S7 and S8), or when analyses were restricted to those reporting continuing education in the last month only (N = 1478; Figure S9).

## Discussion

The most important result arising from this matched longitudinal study is that long-term cognitive trajectories did not improve following mid-to-late life continuing education when compared with matched controls who did not undertake continuing education. This was the case regardless of age, sex, or cognitive status. The results suggest that participation in continuing education during mid-to-late life may not confer a long-term cognitive benefit.

The findings stand in contrast with previous observational studies^6^, ^7^, ^8^, ^9^^,^^20^^,^^21^ and one small-scale (N = 459) trial with non-random allocation in which participants self-enrolled in continuing education,^10^ which have reported associations between adult education and better cognitive performance or reduced dementia risk. A key methodological limitation of this body of literature is the issue of reverse causality: individuals with poorer cognitive function or those experiencing accelerated cognitive decline may be less likely to pursue formal education or training later in life. We addressed this limitation by selecting a control group that was closely matched to the continuing education group on multiple characteristics, including cognitive function prior to participation in continuing education. We further showed that in our analytic sample, cognitive performance at the time of continuing education or the equivalent time point for controls was only weakly correlated with subsequent rate of cognitive decline. We thus demonstrated that when reverse causality is accounted for, continuing education does not appear to improve memory or fluency trajectories.

It was notable that the multiple continuing education group had less cognitive decline than is generally seen in their age group.^22^ However, matched controls showed the same better-than-expected trajectory. This pattern suggests that both groups were healthier and cognitively higher-functioning than the population average at enrolment, consistent with selective participation in continuing education rather than a causal effect of continuing education itself. It is nonetheless possible that the matched control group—selected to resemble a cognitively high-performing continuing education group—were themselves engaged in other cognitively stimulating activities, attenuating any observable benefit of continuing education. However, the absence of effect was also observed among individuals with evidence of cognitive impairment, suggesting this explanation is unlikely to fully account for the findings.

In sensitivity analyses, we observed a modest stepwise increase in memory scores among participants with a single report of continuing education. However, this benefit appeared to be transient: the continuing education group declined slightly faster thereafter, such that by the end of follow-up their performance was indistinguishable from controls. Thus, any short-term improvement associated with participation did not translate into sustained differences in long-term trajectories.

There are several strengths of this study. Our study design had quasi-experimental elements, where we selected matched controls and examined trajectories before and after continuing education, strengthening causal inference and addressing the issue of reverse causality more effectively than previous studies. We used data drawn from a largescale nationally representative study population followed up over more than two decades, enhancing generalisability and allowing us to examine long-term cognitive trajectories. We matched on follow-up duration, reducing issues of differential attrition or mortality affecting the results; while this may bias findings toward a spurious association, we observed a null result regardless. Finally, we mitigated the impact of participation in continuing education occurring before the study by matching on cognitive performance before continuing education.

There are several limitations to consider regarding generalisability, which pose areas for future research. Individuals engaging in continuing education had higher socioeconomic position and better overall health than the general population, potentially limiting generalisability to less healthy populations. While our sensitivity analysis suggested little evidence of a difference in results by education level, it remains possible that continuing education could confer a cognitive benefit among individuals with worse general health. Because continuing education participants typically have higher baseline cognitive performance, matching required excluding those at the very high end of performance (and their lowest-performing non-continuing education counterparts). This improves comparability but means our results are most relevant to the middle range of the cognitive distribution. However, in subgroup analyses restricted to participants with evidence of cognitive impairment, we found no benefit of continuing education, suggesting that our findings also extend to individuals at the lower end of cognitive performance. Though ELSA participants who move into institutional settings during follow-up continue to be interviewed, individuals who are institutionalised at baseline are excluded, which may reduce generalisability among these populations. ELSA is 95% white, reflecting demographic characteristics of over 50s in England during the study period,^23^ and results may be generalisable to similar study populations. Nevertheless, though our study draws on data from England only, the challenges of dementia prevention and the emphasis on modifiable risk factors are shared across Europe, making these findings relevant for policy discussions in the region.

Other limitations concern exposure definition, cognitive testing, and sample size considerations. Because participants reporting continuing education at baseline were excluded to allow a defined pre-exposure period, our findings apply to continuing education initiated during follow-up. It remains possible that sustained continuing education beginning earlier in adulthood has a cumulative cognitive effect. We examined memory and fluency only, as these are the cognitive tests administered at nearly all waves of ELSA that are not strongly affected by ceiling effects. These are cognitive domains that are key for day-to-day function and are highly relevant to cognitive ageing and dementia diagnosis; however, results may not generalise to other important cognitive domains such as executive function, which may be amenable to lifestyle interventions.^24^ Finally, while our sample size provided good power to detect moderate differences, power calculations indicate that small effects could not be excluded.

Focusing on interventions supported by robust evidence of cognitive benefits is essential to ensure that efforts to promote cognitive health are both effective and equitable. While adult education might offer short-term cognitive engagement and delivers well-established social, psychological, and economic benefits,^25^ our findings suggest it is unlikely to alter the long-term trajectory of memory or fluency decline. This study addresses age-related cognitive decline which is not synonymous with disease-related decline, though they may share common determinants. Current prevention frameworks^3^^,^^26^ consider modification of cognitive ageing to be part of dementia-prevention strategies, but further research will be needed to establish whether the present findings translate into meaningful implications for dementia risk. Our study therefore suggests that public health messaging should avoid framing continuing education as a dementia-prevention strategy until stronger evidence is available.

In contrast to continuing education, high-quality education in early life has consistent and robust associations with better cognitive outcomes across the life course,^3^^,^^27^^,^^28^ through pathways such as socioeconomic attainment, healthier lifestyles, and potentially brain resilience.^29^^,^^30^ As governments across the WHO Europe region strengthen strategies to address the growing dementia burden, our results suggest that investment in equitable access to early-life education should remain the clear priority. Later-life education may offer social, psychological, and short-term cognitive benefits, but further research is needed before its long-term role in cognitive health can be established.

## Contributors

MB and AS conceptualised the study. MB and SS were responsible for methodology. MB was responsible for the formal analysis. MB and AS were responsible for data curation. MB wrote the original draft. MB, SS, FB, JG, and AS contributed to review and editing. MB was responsible for visualisation. AS was responsible for supervision and funding acquisition. MB and AS accessed and verified the data. All authors had full access to the data and accept responsibility to submit for publication.

## Data sharing statement

ELSA data are available to researchers after registration with the UK data service at https://beta.ukdataservice.ac.uk/datacatalogue/series/series?id=200011.

## Declaration of interests

Séverine Sabia reports research funding to her institution from the European Union (ERC grant number 101043884), Fondation Alzheimer, and Fondation Vaincre Alzheimer.

Jessica Gong reports research funding to her institution from the U.S. National Institute on Aging, National Institutes of Health (R01AG030153 and R01AG17644). She also reports personal payments from Genome Québec (Genomics Integration Program grant review), from Imperial College London (honoraria for the MESSAGE short course “Integrating sex and gender in health research and policy”), and from the Multidisciplinary Digital Publishing Institute (MDPI Travel Grant).

All other authors declare no competing interests.
