# Supplementary material for: Cognitive decline before and after mid-to-late-life continuing education in England: a matched longitudinal analysis of a prospective cohort study
Source: Lancet Reg Health Eur. 2025 Oct 29;60:101513. doi: 10.1016/j.lanepe.2025.101513 (PMC12605658; doi:10.1016/j.lanepe.2025.101513)
Supplement: Supplementary Materials [file mmc1.pdf]

Supplementary materials

Cognitive decline before and after mid-to-late-life continuing education in England: a matched longitudinal analysis of a prospective cohort study

Contents

1. Supplementary methods. 3

Methods S1. CES-D Scale. 3

Methods S2. Matching. 4

Methods S3. Stata code for main analysis. 7

Methods S4. Additional analyses. 8

2. Supplementary tables. 10

Table S1. ELSA participants excluded from the analysis compared with those included in the analytic sample. 10

Table S2. ELSA participants in continuing education groups excluded during matching compared with those included in the analytic sample. 11

Table S3. Number of waves of participation in the single continuing education group and controls. 12

Table S4. Number of waves of participation in the multiple continuing education group and controls. 13

Table S5. Coefficients for interaction for models with $aget0$ interactions. 14

Table S6. Coefficients for interaction for models with sex interactions. 15

Table S7. Coefficients for interaction in models with interactions with baseline education level. 16

Table S8. Discontinuity terms for models allowing step change in cognitive performance at $t0$ 17

3. Supplementary figures. 18

Figure S1. Spline-based trajectories of memory scores relative to $t0$. 18

Figure S2. Spline-based trajectories of fluency scores relative to $t0$. 19

Figure S3. Flowchart of sample selection. 20

Figure S4. Average cognitive trajectories before and after continuing education in the single continuing education group and controls additionally accounting for practice effects (N=1,442). 21

Figure S5. Average cognitive trajectories before and after continuing education in the multiple continuing education group and controls additionally accounting for practice effects (N=2,696). 22

Figure S6. Average cognitive trajectories before and after continuing education when the continuing education group gained a degree qualification (N=1,102). 23

Figure S7. Average cognitive trajectories before and after continuing education when analytic sample is restricted to participants with evidence of cognitive impairment (N=920). 24

Figure S8. Average cognitive trajectories before and after continuing education when analytic sample is restricted to participants without evidence of cognitive impairment (N=1,790). 25

Figure S9. Average cognitive trajectories before and after continuing education when analytic sample is restricted to participants reporting continuing education in the last month (N=1,478). 26

4. References. 27

1. Supplementary methods.

Methods S1. CES-D Scale.

Depressive symptoms were assessed using the 8-item Center for Epidemiologic Studies Depression (CES-D) Scale,[1] which requires participants to report whether they often (yes or no): 1) feel depressed; 2) feel everything is an effort; 3) have restless sleep; 4) are happy; 5) feel lonely; 6) feel sad; 7) cannot get going; and 8) enjoy life. One point was scored for each item with ‘happy’ and ‘enjoyed life’ reverse scored. A higher score indicates more depressive symptoms (range: 0-8).

Methods S2. Matching.

We used coarsened exact matching (CEM), a method which is robust to measurement error, requires fewer assumptions than other matching methods, and does not require the iterative checking process to guarantee balance necessary for other methods.[2] Coarsened exact matching was implemented using the *cem* package[3] in Stata with the k-to-k procedure used to produce an equal number of participants in the continuing education and control groups. In this procedure, individuals in the continuing education group are assigned to a matching stratum with controls based on age at baseline, sex, education level, follow up duration, and mean standardised cognitive score at baseline. Controls are then randomly dropped to yield an equal numbers in the continuing education and control groups in each stratum. These matching variables were chosen to maximise the comparability of exposure groups whilst minimising data loss, as each additional matching variable leads to more participants dropped from the analysis due to not having matches. For this reason, we adjusted for other confounders rather than including them as matching variables.

We used the k-to-k procedure to simplify analyses and reporting of the results. For valid causal inference following CEM, each matching stratum must have an equal number of individuals in each exposure group (i.e., the number in the continuing education group must equal the number of controls in each stratum).[2] To use all matched participants for modelling, it is therefore necessary to apply model weights to account for differences in the numbers in each exposure group. These weights are produced following the CEM procedure. However, we include other covariates in the model in addition to the matching variables, due to variable coarsening and to account for other imbalances between the continuing education and control groups. As a result, it is no longer clear how the model should be weighted to ensure balance. Using the k-to-k procedure avoids these complications as it does not require the use of weights for valid causal inference.

For mean cognitive score, where there were no obvious cut points based on a priori knowledge, cut points for matching were determined using Sturges' rule,[4] which led us to divide cognitive scores into 15 bins of 0.34 standard deviations (range: -2.85 to 2.32). For all other matching criteria, cut points were defined in Table S2.1 below.

Table S2.1. Cut points used for coarsened exact matching.

Variable	Cut points
Age at baseline	Five-year age groups from 50-54 to 85-89
Sex	Male<br>Female
Educational qualifications	Less than high school<br>High school<br>Above high school
Follow-up duration	Two-year groups from 1-21 years

We selected two separate control groups using this matching procedure. The first control group was matched to the multiple continuing education group (those reporting more than one instance of continuing education during the follow up period). The second control group was matched to the single continuing education group (those reporting just one instance of continuing education during the follow-up period). We examined these groups separately because individuals regularly engaging in continuing education differ from those engaging in just a single instance of continuing education. Both control groups were selected from the general ELSA study population, and individuals could be included in either or both control groups.

The change in absolute standardised mean difference in matching variables before and after matching demonstrating the improvement in balance is demonstrated in the following Love plots (Figure S2.1).

Figure S2.1. Love plots showing balance in matched and unmatched samples.

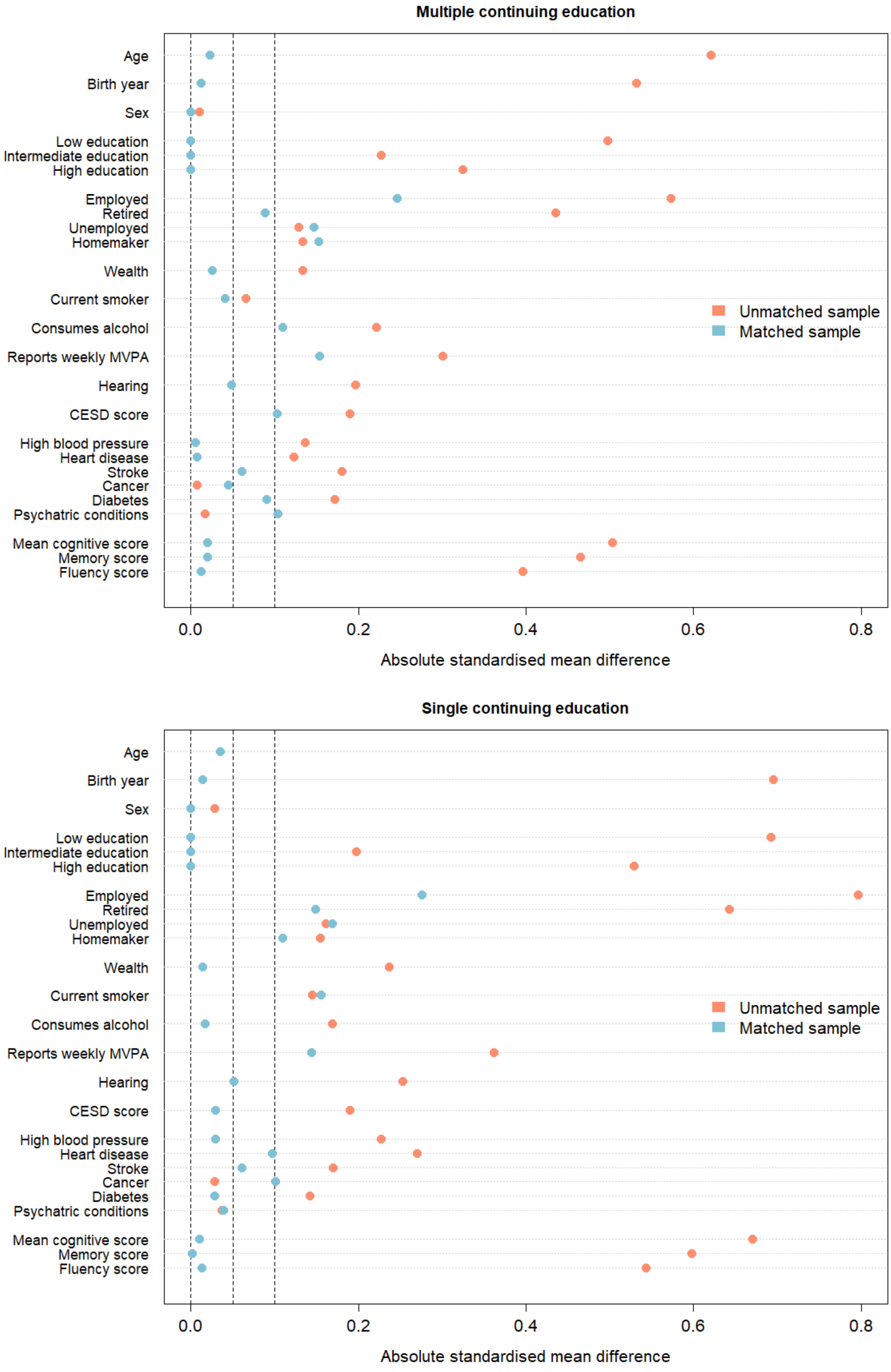

Absolute standardised mean difference 0.00, 0.05, and 0.10 indicated using dashed lines.

Methods S3. Stata code for main analysis.

```
*Memory
mixed ztr20 ///
	///
	i.ever_ae##c.time1 i.ever_ae##c.time2 ///
	///
	c.age_ae65##c.time1 c.age_ae65##c.time2 ///
	///
	c.time2##c.time2 ///
	///
	i.ragender c.rabyear i.raeducl0 i.lbf0 c.zwealth0 ///
	i.rsmoken0 i.rdrink0 i.pa0 c.cesd0 c.rhearing0 ///
	i.rstroke0 i.rhearte0 i.rcancre0 i.rdiabe0 i.rhibpe0 ///
	///
	|| id: time1 time2, cov(uns)

est save ztr20_est, replace

*Fluency
mixed zverbf ///
	///
	i.ever_ae##c.time1 i.ever_ae##c.time2 ///
	///
	c.age_ae65##c.time1 c.age_ae65##c.time2 ///
	///
	i.ragender c.rabyear i.raeducl0 i.lbf0 c.zwealth0 ///
	i.rsmoken0 i.rdrink0 i.pa0 c.cesd0 c.rhearing0 ///
	i.rstroke0 i.rhearte0 i.rcancre0 i.rdiabe0 i.rhibpe0 ///
	///
	|| id: time1 time2, cov(uns)

 est save zverbf_est, replace

*Marginal estimates for plots
foreach out in ztr20 zverbf {
	est use `out'_est
	est esample: if !missing(`out')
	qui: margins ever_ae, ///
		at(time2=0 time1=(-4(1)-1 -0.0001)) ///
		///
		at(time1=0 time2=(0(1)8)) ///
		post
	est save margins_`out', replace
}
```

Methods S4. Additional analyses.

We first examined whether there was evidence for variation in results by age_{t0} by additionally including three-way interactions between education group, time terms, and age_{t0} in models. We then examined evidence for variation in results by sex by additionally including two- and three-way interactions between sex, education group, and time terms in models. Finally, we examined whether results differed by baseline education level by including two- and three-way interactions between baseline education level, education group, and time terms.

Next, as 'formal education and training' covers a wide range of activities of varying intensity and duration, we wanted to determine whether there was an impact on cognitive decline of more structured and sustained formal education specifically. In this analysis, we restricted the continuing education group to participants who reported gaining an additional educational qualification following continuing education. Education level was based on the National Vocational Qualifications framework,[5] a structured system for assessing and recognising work-based skills and competencies across various levels and industries, aligned with national occupational standards. If an individual moved up by an NVQ level, they were considered to be part of the continuing education group. Controls in this analysis neither reported continuing education nor changing educational qualifications during the follow-up period.

**NVQ Level**	**Description**
Level 1	Competence, which involves the application of knowledge and skills in the performance of a range of varied work activities most of which may be routine and predictable.
Level 2	Competence, which involves the application of knowledge and skills in a significant range of varied work activities, performed in a variety of contexts. Some of the activities are complex or non-routine, and there is some individual responsibility or autonomy. Collaboration with others, perhaps through membership of a work group or team, may often be a requirement.
Level 3	Competence, which involves the application of knowledge and skills in a broad range of varied work activities performed in a wide variety of contexts and most of which are complex and non-routine. There is considerable responsibility and autonomy, and control or guidance of others is often required.
Level 4	Competence, which involves the application of knowledge and skills in a broad range of complex, technical, or professional work activities performed in a wide variety of contexts and with a substantial degree of personal responsibility and autonomy.

	Responsibility for the work of others and the allocation of resources is often present.
Level 5	Competence, which involves the application of skills and a significant range of fundamental principles and complex techniques across a wide and often unpredictable variety of contexts. Very substantial personal autonomy and often a significant responsibility for the work of others and for the allocation of substantial resources feature strongly, as do personal accountabilities for analysis and diagnosis, design, planning, execution and evaluation.

Reproduced from the UK Government Department for Business, Innovation, and Skills.

We also performed analyses separately in those who did and did not show evidence of cognitive impairment, where cognitive impairment was defined as scoring 1.5 standard deviations below their five-year age and education group in at least one cognitive domain (a threshold commonly used when clinical assessment is not possible[6]) or self-reporting a dementia diagnosis. To be considered for these analyses, participants could report dementia diagnosis or meet criteria for cognitive impairment any time during the study period.

2. Supplementary tables.

Table S1. ELSA participants excluded from the analysis compared with those included in the analytic sample.

	**Excluded** N=17,286	**Included** N=3,906	*P-value*
Age in years, mean (SD)	62.4 (10.1)	57.6 (7.2)	<0.0001
Birth year, mean (SD)	1944.6 (14.1)	1947.3 (9.5)	<0.0001
Sex			
Male	8016 (46.4)	1743 (44.6)	
Female	9270 (53.6)	2163 (55.4)	0.050
Education level			
Below high school	6973 (44.2)	958 (24.5)	
High school	6387 (40.5)	2129 (54.5)	<0.0001
Above high school	2413 (15.3)	819 (21.0)	
Labour force status			
Employed	7605 (44.1)	2307 (59.1)	
Retired	6827 (39.5)	999 (25.6)	<0.0001
Unemployed/disabled	1404 (8.1)	281 (7.2)	
Homemaker/other	1428 (8.3)	319 (8.2)	
Standardised wealth, mean (SD)	-0.02 (1.1)	0.11 (1.2)	<0.0001
Current smoker	2930 (18.3)	666 (17.1)	0.079
Currently consumes alcohol	13316 (88.1)	3611 (92.4)	<0.0001
Reports weekly MVPA	13019 (76.2)	3308 (84.7)	<0.0001
Self-rated hearing			
Excellent	4034 (23.4)	1069 (27.4)	
Very good	4703 (27.2)	1118 (28.6)	<0.0001
Good	5143 (29.8)	1136 (29.1)	
Fair	2572 (14.9)	496 (12.7)	
Poor	823 (4.8)	87 (2.2)	
CES-D score, mean (SD)	1.6 (2.0)	1.4 (1.9)	<0.0001
Diagnosis of:			
High blood pressure	5680 (32.9)	1063 (27.2)	<0.0001
Heart disease	2634 (15.3)	394 (10.1)	<0.0001
Stroke	620 (3.6)	45 (1.2)	<0.0001
Cancer	1054 (6.1)	203 (5.2)	0.034
Diabetes	1399 (8.1)	170 (4.4)	<0.0001
Psychiatric conditions	1384 (8.0)	333 (8.5)	0.30
Cognitive scores, mean (SD)			
Standardised memory	-0.11 (1.0)	0.25 (0.82)	<0.0001
Standardised fluency	-0.12 (1.0)	0.18 (0.83)	<0.0001
Standardised mean	-0.11 (1.0)	0.25 (0.76)	<0.0001

N (%) shown unless otherwise indicated.

Abbreviations: SD, standard deviation; MVPA, moderate-to-vigorous physical activity; CES-D, Center for Epidemiologic Studies Depression.

Table S2. ELSA participants in continuing education groups excluded during matching compared with those included in the analytic sample.

	**Excluded** N=222	**Included** N=2,069	*P-value*
Age in years, mean (SD)	59.2 (8.2)	57.4 (7.1)	0.0016
Birth year, mean (SD)	1945.5 (10.3)	1947.4 (9.4)	0.0090
Sex			
Male	106 (47.7)	922 (44.6)	
Female	116 (52.3)	1147 (55.4)	0.40
Education level			
Below high school	25 (11.3)	490 (23.7)	
High school	52 (23.4)	1127 (54.5)	<0.0001
Above high school	145 (65.3)	452 (21.8)	
Labour force status			
Employed	120 (54.1)	1360 (65.7)	
Retired	79 (35.6)	472 (22.8)	0.00038
Unemployed/disabled	9 (4.1)	107 (5.2)	
Homemaker/other	14 (6.3)	130 (6.3)	
Standardised wealth, mean (SD)	0.28 (1.0)	0.13 (1.1)	0.034
Current smoker	25 (11.3)	318 (15.4)	0.13
Currently consumes alcohol	211 (95.0)	1935 (93.5)	0.46
Reports weekly MVPA	198 (89.2)	1807 (87.3)	0.49
Self-rated hearing			
Excellent	71 (32.0)	589 (28.5)	
Very good	54 (24.3)	578 (27.9)	0.65
Good	66 (29.7)	617 (29.8)	
Fair	28 (12.6)	242 (11.7)	
Poor	3 (1.4)	43 (2.1)	
CES-D score, mean (SD)	1.1 (1.6)	1.3 (1.8)	0.15
Diagnosis of:			
High blood pressure	54 (24.3)	559 (27.0)	0.43
Heart disease	1 (0.5)	20 (1.0)	0.69
Stroke	19 (8.6)	197 (9.5)	0.73
Cancer	13 (5.9)	119 (5.8)	>0.99
Diabetes	11 (5.0)	74 (3.6)	0.40
Psychiatric conditions	17 (7.7)	155 (7.5)	>0.99
Cognitive scores, mean (SD)			
Standardised memory	0.43 (1.1)	0.27 (0.82)	0.040
Standardised fluency	0.61 (1.3)	0.20 (0.82)	<0.0001
Standardised mean	0.55 (1.3)	0.27 (0.75)	0.0012

N (%) shown unless otherwise indicated.
Abbreviations: SD, standard deviation; MVPA, moderate-to-vigorous physical activity; CES-D, Center for Epidemiologic Studies Depression.

Table S3. Number of waves of participation in the single continuing education group and controls.

**Number of waves**	N (%)	
	Controls	*Continuing education*
1	721 (100)	721 (100)
2	721 (100)	721 (100)
3	701 (97.2)	721 (100)
4	660 (91.5)	691 (95.8)
5	619 (85.9)	644 (89.3)
6	561 (77.8)	580 (80.4)
7	480 (66.6)	517 (71.7)
8	372 (51.6)	395 (54.8)
9	285 (39.5)	308 (42.7)
10	178 (24.7)	185 (25.7)

"Number of waves" refers to the number of ELSA interviews attended since baseline (i.e., visit count). Percentages are relative to the baseline sample size in each group.

Table S4. Number of waves of participation in the multiple continuing education group and controls.

**Number of waves**	**N (% remaining)**	
	Controls	*Continuing education*
1	1348 (100)	1348 (100)
2	1348 (100)	1348 (100)
3	1187 (88.1)	1218 (90.4)
4	1068 (79.2)	1099 (81.5)
5	946 (70.2)	965 (71.6)
6	816 (60.5)	827 (61.4)
7	702 (52.1)	704 (52.2)
8	514 (38.1)	527 (39.1)
9	376 (27.9)	394 (29.2)
10	221 (16.4)	244 (18.1)

"Number of waves" refers to the number of ELSA interviews attended since baseline (i.e., visit count). Percentages are relative to the baseline sample size in each group.

Table S5. Coefficients for interaction for models with age_{t0} interactions.

	Coefficient (95% CI)	P-value
**Memory**		
Single continuing education		
Continuing education x pre-time x age_{t0}	-0.000 (-0.001-0.001)	0.51
Continuing education x post-time x age_{t0}	-0.001 (-0.002-0.000)	0.31
Multiple continuing education		
Continuing education x pre-time x age_{t0}	-0.000 (-0.003-0.002)	0.71
Continuing education x post-time x age_{t0}	-0.000 (-0.001-0.001)	0.65
**Fluency**		
Single continuing education		
Continuing education x pre-time x age_{t0}	-0.001 (-0.001-0.002)	0.40
Continuing education x post-time x age_{t0}	-0.000 (-0.002-0.001)	0.56
Multiple continuing education		
Continuing education x pre-time x age_{t0}	-0.001 (-0.004-0.002)	0.48
Continuing education x post-time x age_{t0}	-0.001 (-0.003-0.000)	0.079

Models adjusted for all lower order interactions, age_{t0}, sex, baseline education level, labour force status, wealth, health behaviours, self-rated hearing, depressive symptoms, and long-term conditions.

Table S6. Coefficients for interaction for models with sex interactions.

	Coefficient (95% CI)	P-value
**Memory**		
Single continuing education		
Continuing education x pre-time x female	-0.005 (-0.022-0.013)	0.62
Continuing education x post-time x female	0.003 (-0.011-0.017)	0.70
Multiple continuing education		
Continuing education x pre-time x female	-0.017 (-0.050-0.015)	0.31
Continuing education x post-time x female	0.006 (-0.007-0.019)	0.38
**Fluency**		
Single continuing education		
Continuing education x pre-time x female	0.019 (-0.001-0.038)	0.062
Continuing education x post-time x female	-0.007 (-0.025-0.011)	0.44
Multiple continuing education		
Continuing education x pre-time x female	0.019 (-0.017-0.056)	0.30
Continuing education x post-time x female*	0.022 (0.004-0.040)	0.019

Models adjusted for all lower order interactions, age_{t0}, sex, baseline education level, labour force status, wealth, health behaviours, self-rated hearing, depressive symptoms, and long-term conditions.

*While a statistically significant difference in post-education fluency slope was observed among women, a similar (non-significant) difference was already present prior to education, and the slope estimates before (β=0.012, 95% CI=-0.015-0.039) and after (β=0.013, 95% CI=0.001-0.024) were nearly identical. This suggests no change in trajectory and is unlikely to reflect a causal effect.

Table S7. Coefficients for interaction in models with interactions with baseline education level.

	Estimate (95% CI)	P-value
**Single continuing education**		
Memory		
Intermediate education x group x pre-time	0.007 (-0.014 to 0.027)	0.51
High education x group x post-time	0.004 (-0.022 to 0.030)	0.76
Intermediate education x group x post-time	-0.005 (-0.022 to 0.011)	0.51
High education x group x post-time	-0.005 (-0.027 to 0.017)	0.66
Fluency		
Intermediate education x group x pre-time	0.004 (-0.017 to 0.026)	0.69
Discontinuity x continuing education	-0.009 (-0.037 to 0.019)	0.54
Intermediate education x group x post-time	-0.015 (-0.036 to 0.005)	0.15
Discontinuity x continuing education	-0.007 (-0.036 to 0.022)	0.62
**Multiple continuing education**		
Memory		
Intermediate education x group x pre-time	0.001 (-0.044 to 0.047)	0.95
High education x group x post-time	-0.007 (-0.056 to 0.042)	0.77
Intermediate education x group x post-time	-0.008 (-0.024 to 0.009)	0.36
High education x group x post-time	-0.004 (-0.024 to 0.015)	0.67
Fluency		
Intermediate education x group x pre-time	-0.027 (-0.077 to 0.022)	0.28
High education x group x post-time	-0.062 (-0.117 to -0.008)	0.024
Intermediate education x group x post-time	-0.017 (-0.040 to 0.006)	0.15
High education x group x post-time	-0.016 (-0.044 to 0.011)	0.24

Group refers to continuing education or control. Reference: low education. Low education is equivalent to below high school, intermediate, high school, and high, above high school. Models adjusted for all lower order interactions, age_{t0}, sex, baseline education level, labour force status, wealth, health behaviours, self-rated hearing, depressive symptoms, and long-term conditions.

Table S8. Discontinuity terms for models allowing step change in cognitive performance at t_0.

	Estimate (95% CI)	P-value
**Single continuing education**		
Memory		
Discontinuity	-0.045 (-0.097 to 0.006)	0.086
Continuing education	0.024 (-0.047 to 0.095)	0.51
Discontinuity x continuing education	0.087 (0.019 to 0.155)	0.012
Fluency		
Discontinuity	-0.010 (-0.064 to 0.044)	0.71
Continuing education	0.002 (-0.076 to 0.080)	0.96
Discontinuity x continuing education	0.031 (-0.043 to 0.105)	0.41
**Multiple continuing education**		
Memory		
Discontinuity	-0.032 (-0.107 to 0.043)	0.41
Continuing education	-0.034 (-0.140 to 0.071)	0.53
Discontinuity x continuing education	0.073 (-0.023 to 0.175)	0.16
Fluency		
Discontinuity	-0.034 (-0.112 to 0.050)	0.43
Continuing education	-0.019 (-0.137 to 0.098)	0.75
Discontinuity x continuing education	0.062 (-0.050 to 0.175)	0.28

Estimate for 'continuing education' indicates difference between continuing education and controls just before t_0. 'Discontinuity' estimate indicates step-change in cognitive performance at t_0 in the control group. 'Discontinuity x continuing education' estimate indicates the difference in the step-change in cognitive performance at t_0 between the continuing education and control groups.

3. Supplementary figures.

Figure S1. Spline-based trajectories of memory scores relative to t_0.

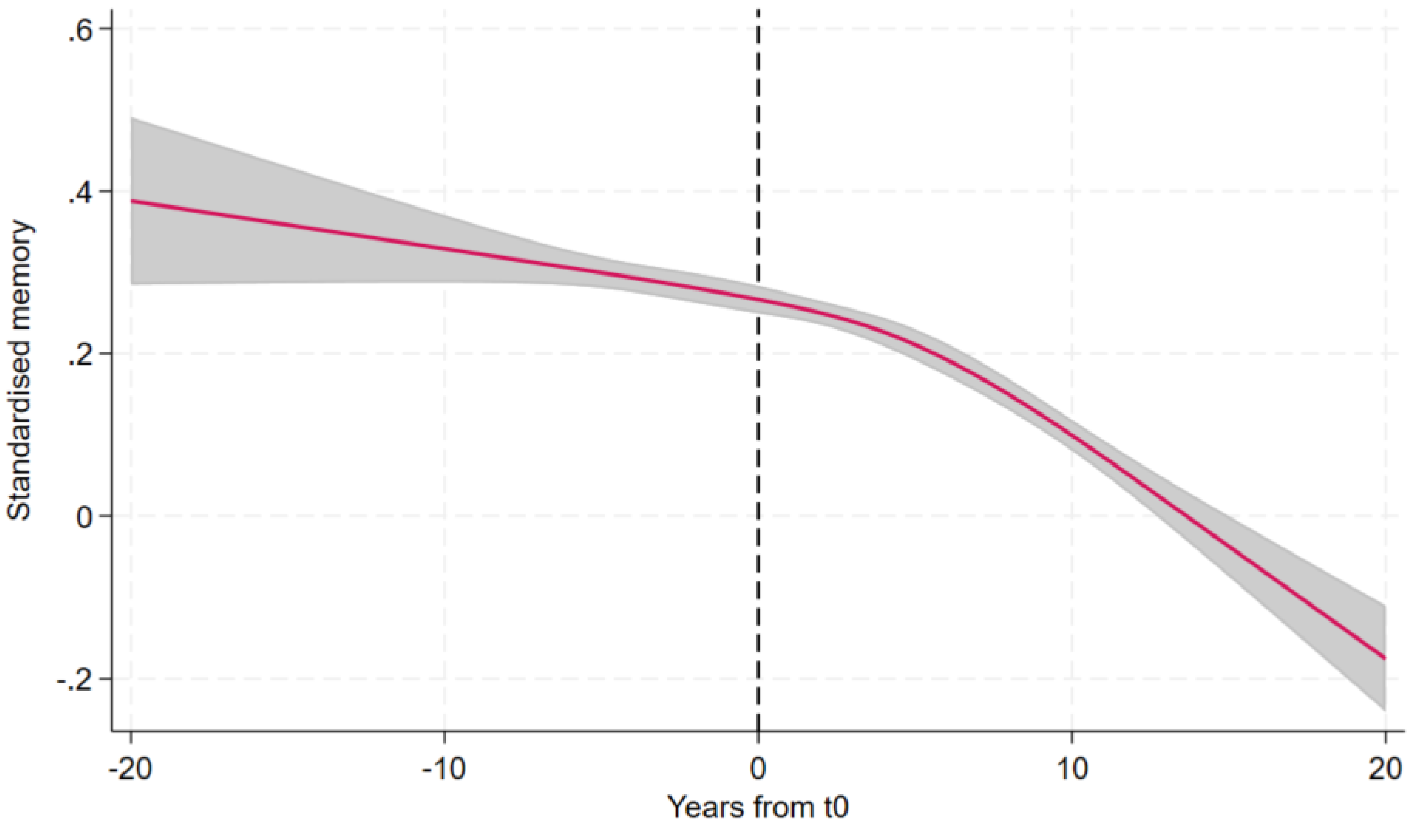

Cubic spline models of standardised memory scores over time from first reported participation in continuing education or the equivalent time point in the controls (t_0). Knots were placed at the 5th, 35th, 65th, and 95th percentiles of follow-up time. Curves are fitted across the full observed range of follow-up (-20 to +20 years), although no single participant contributes observations across the entire span. Within the median follow-up period (-4 to +8 years), the trajectory is well approximated by the piecewise polynomial specification used in the main analyses.

Figure S2. Spline-based trajectories of fluency scores relative to t_0.

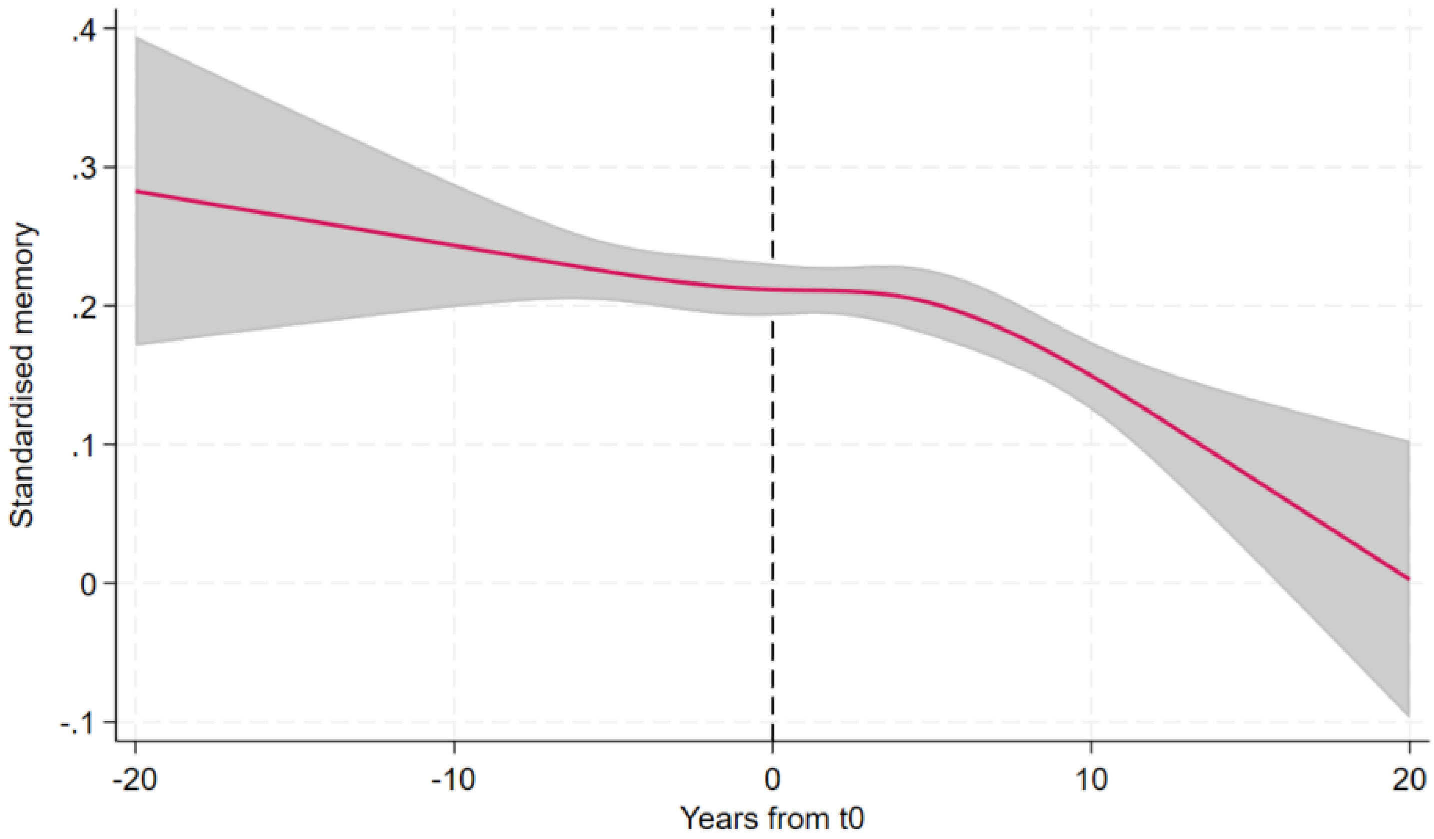

Cubic spline models of standardised fluency scores over time from first reported participation in continuing education or the equivalent time point in the controls (t_0). Knots were placed at the 5th, 35th, 65th, and 95th percentiles of follow-up time. Curves are fitted across the full observed range of follow-up (-20 to +20 years), although no single participant contributes observations across the entire span. Within the median follow-up period (-4 to +8 years), the trajectory is well approximated by the piecewise polynomial specification used in the main analyses.

Figure S3. Flowchart of sample selection.

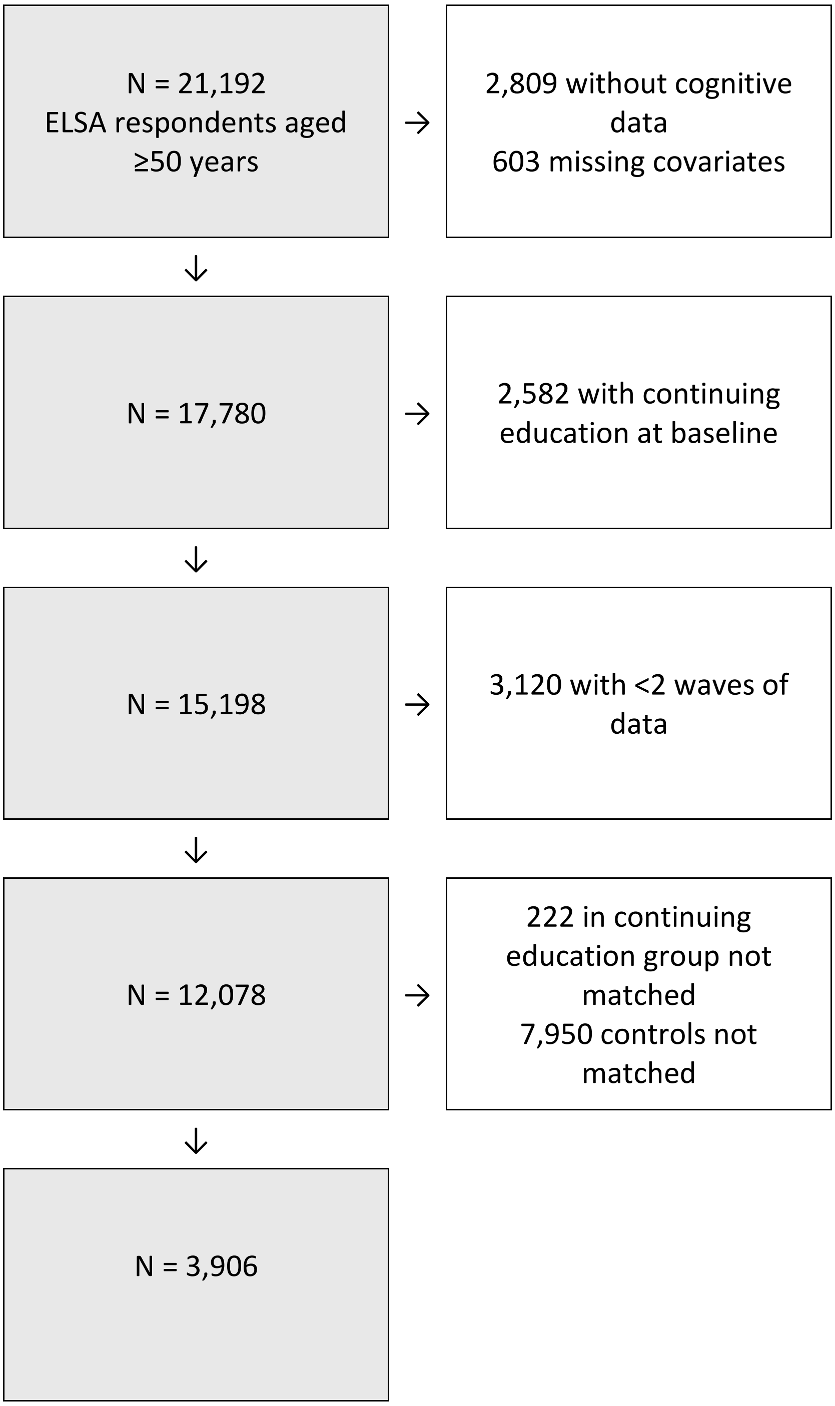

Figure S4. Average cognitive trajectories before and after continuing education in the single continuing education group and controls additionally accounting for practice effects (N=1,442).

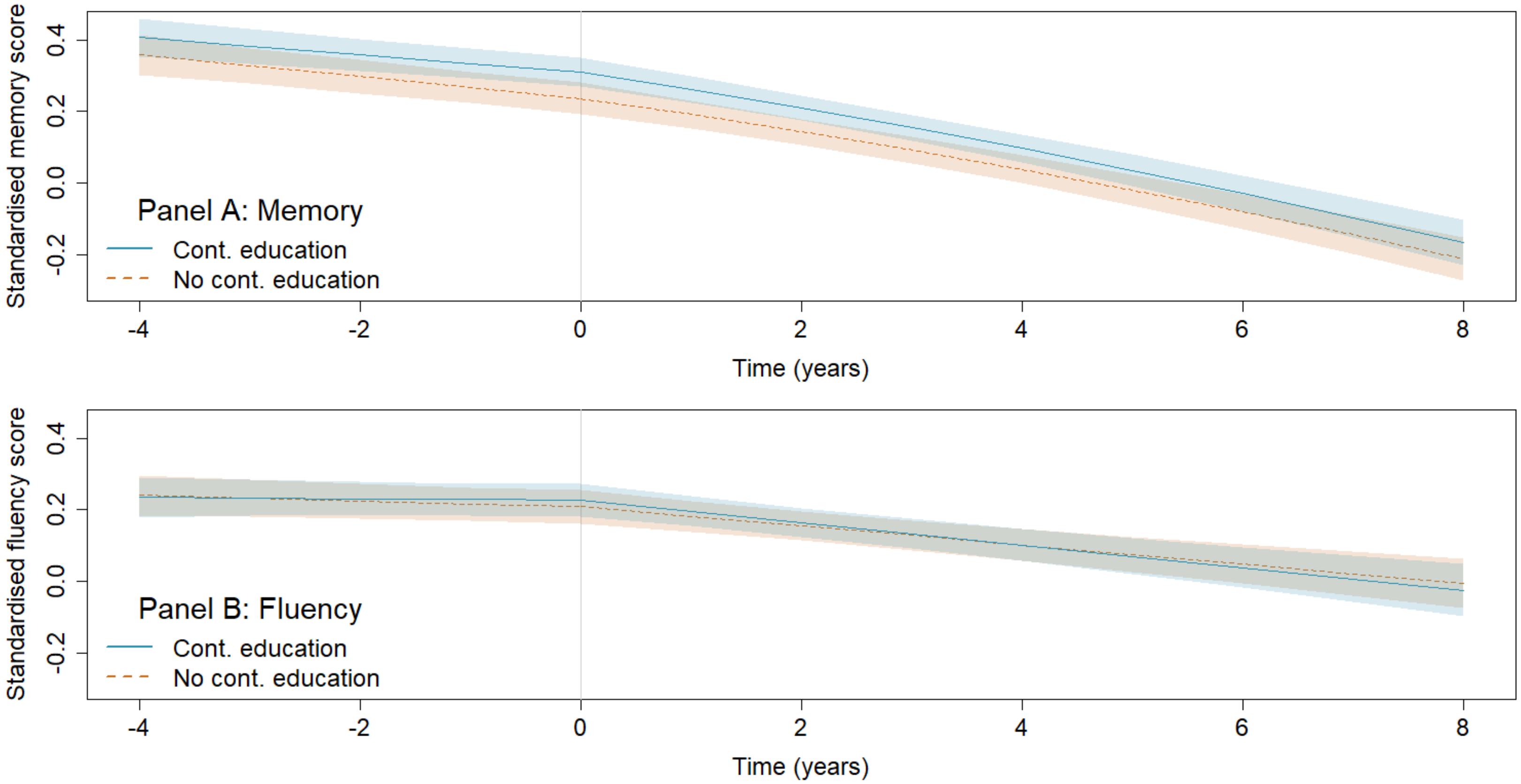

Predicted cognitive scores averaged over the observed covariate distribution in the analytic sample, shown over the median follow-up periods of four years before and eight years after continuing education (time = 0) or the equivalent time interval in the control group. The continuing education group includes 721 participants who reported just one wave of continuing education during the follow-up period. Models were adjusted for age at time = 0, sex, baseline education level, labour force status, wealth, health behaviours, self-rated hearing, depressive symptoms, long-term conditions, and practice effect (round of cognitive assessment).

Figure S5. Average cognitive trajectories before and after continuing education in the multiple continuing education group and controls additionally accounting for practice effects (N=2,696).

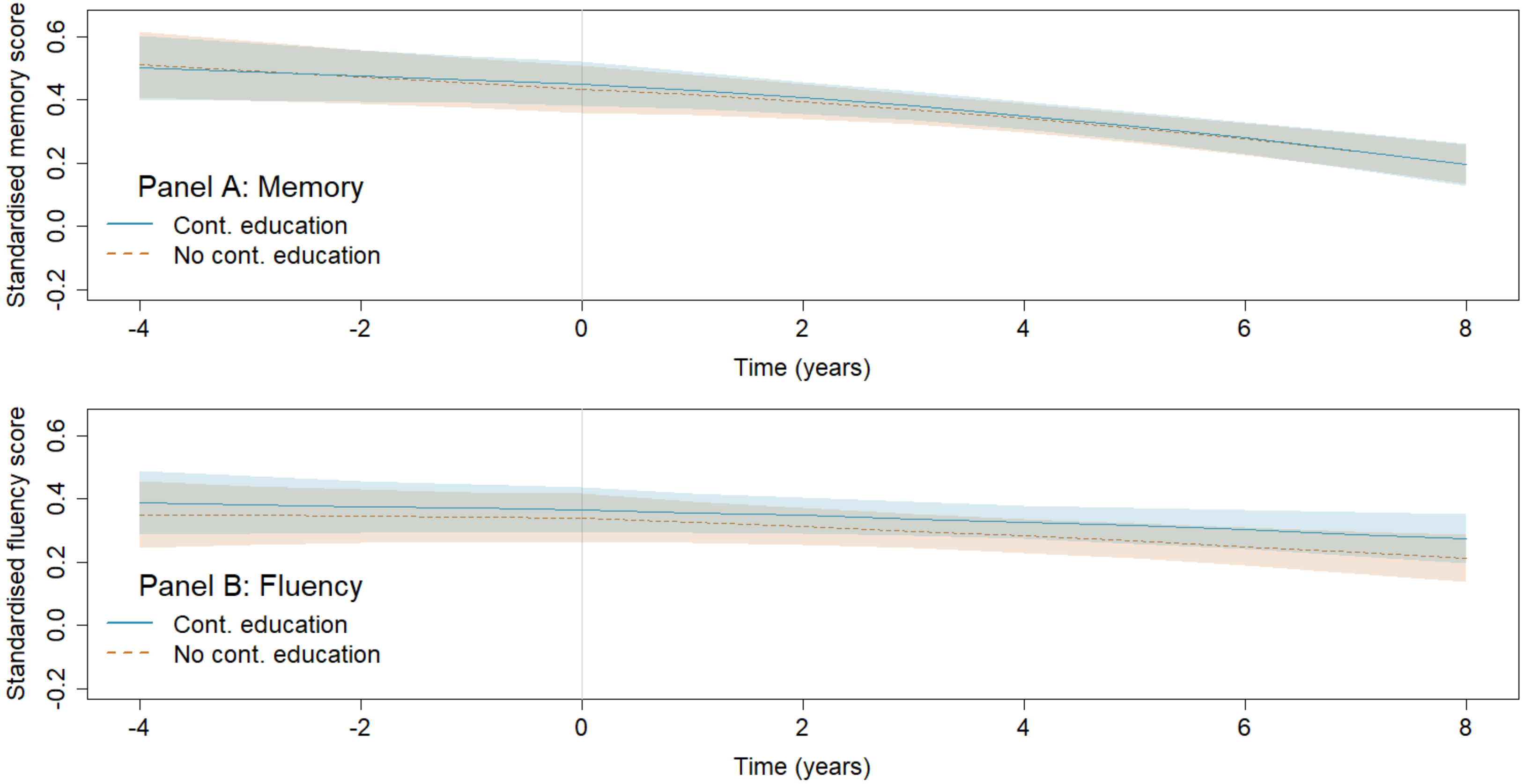

Predicted cognitive scores averaged over the observed covariate distribution in the analytic sample, shown over the median follow-up periods of four years before and eight years after continuing education (time = 0) or the equivalent time interval in the control group. The continuing education group includes 721 participants who reported just one wave of continuing education during the follow-up period. Models were adjusted for age at time = 0, sex, baseline education level, labour force status, wealth, health behaviours, self-rated hearing, depressive symptoms, long-term conditions, and practice effect (round of cognitive assessment).

Figure S6. Average cognitive trajectories before and after continuing education when the continuing education group gained a degree qualification (N=1,102).

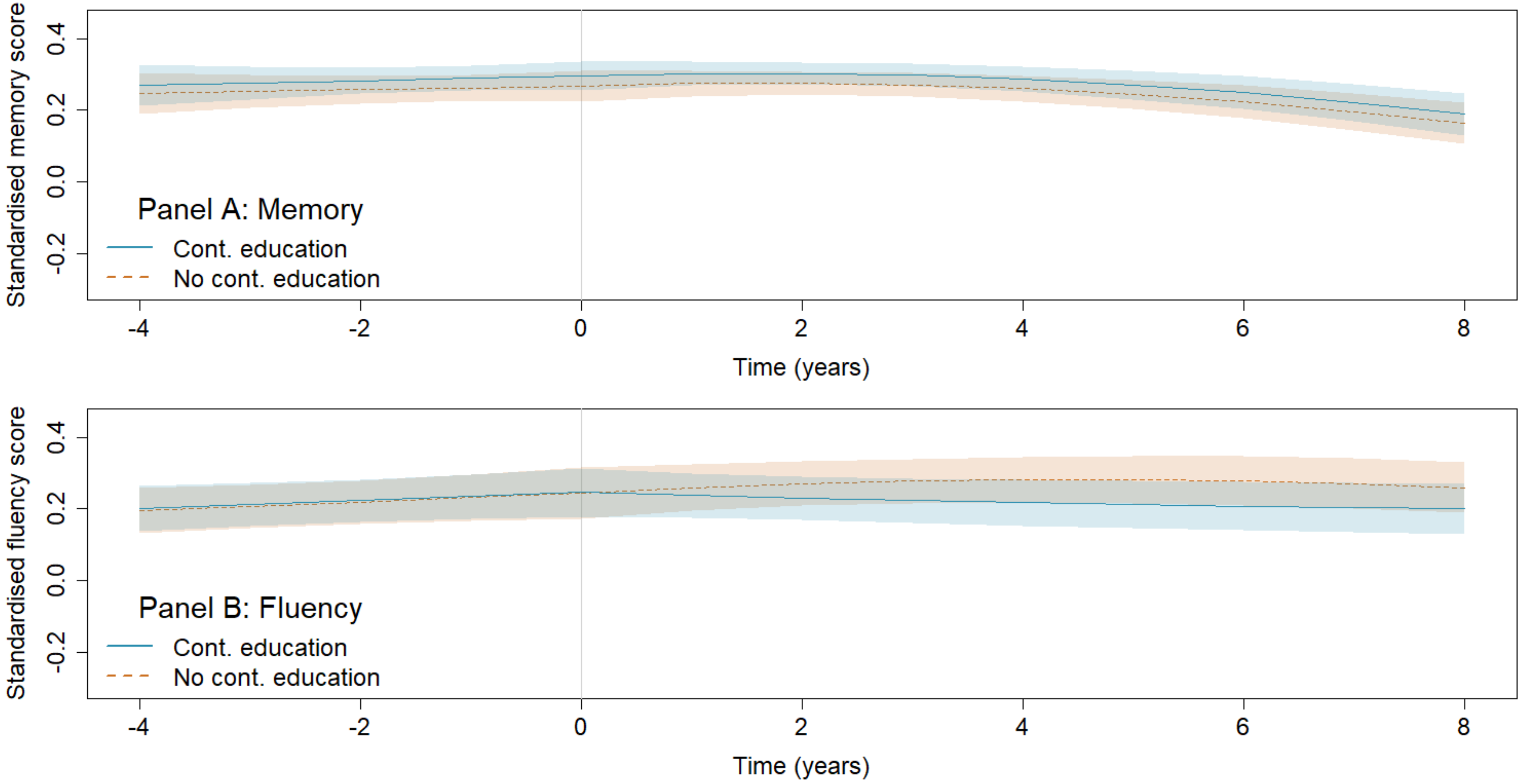

Predicted cognitive scores averaged over the observed covariate distribution in the analytic sample, shown over the median follow-up periods of four years before and eight years after continuing education (time = 0) or the equivalent time interval in the control group. The continuing education group is restricted to N=551 participants reporting gaining a National Vocational Qualifications framework level after continuing education. Time = 0 is time at reporting first continuing education. Models were adjusted for age at time = 0, sex, baseline education level, labour force status, wealth, health behaviours, self-rated hearing, depressive symptoms, and long-term conditions.

Figure S7. Average cognitive trajectories before and after continuing education when analytic sample is restricted to participants with evidence of cognitive impairment (N=920).

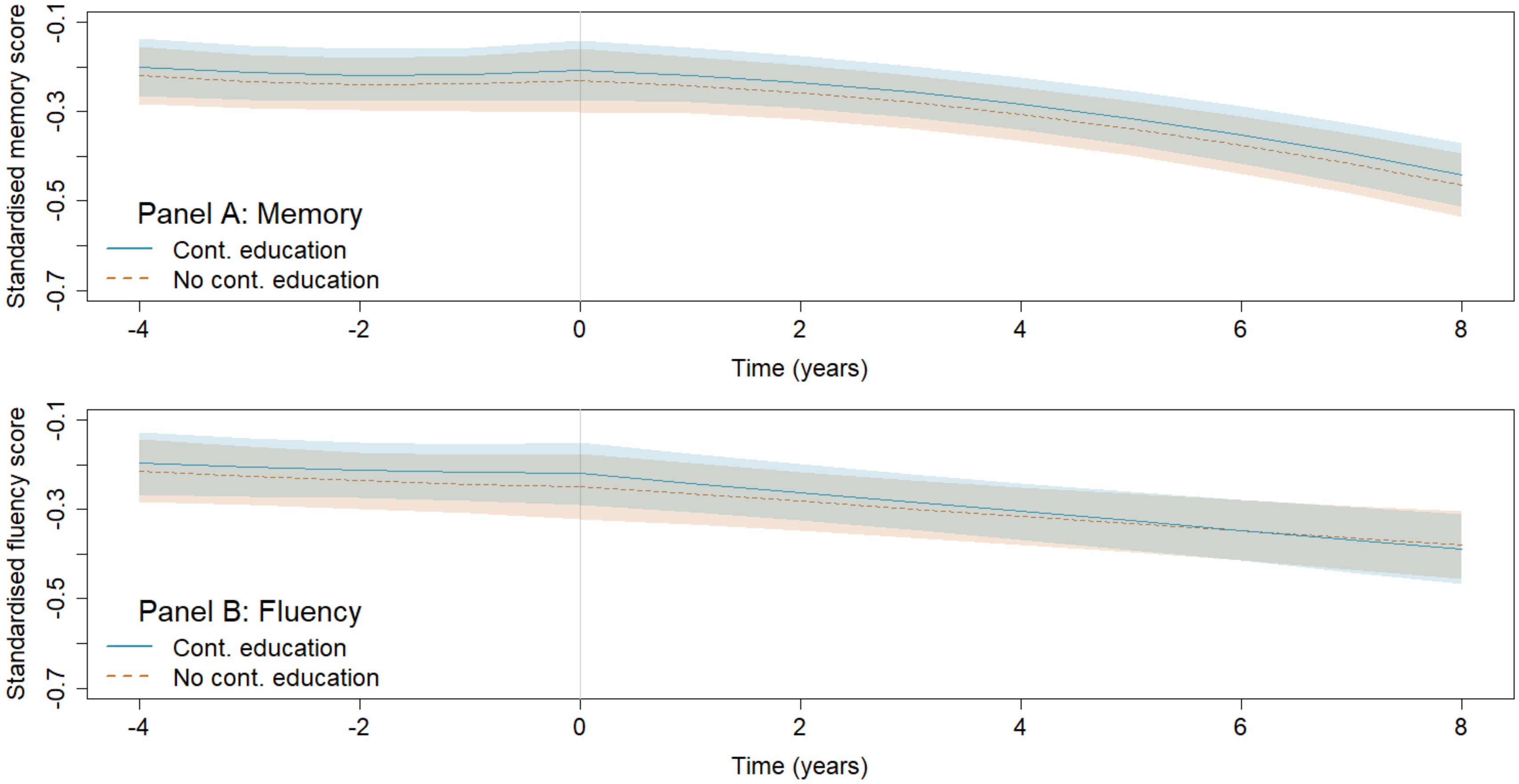

Predicted cognitive scores averaged over the observed covariate distribution in the analytic sample, shown over the median follow-up periods of four years before and eight years after continuing education (time = 0) or the equivalent time interval in the control group. Analyses are restricted to those with cognitive scores suggesting cognitive impairment or reporting a dementia diagnosis (Methods S3). Models were adjusted for age at time = 0, sex, baseline education level, labour force status, wealth, health behaviours, self-rated hearing, depressive symptoms, and long-term conditions.

Figure S8. Average cognitive trajectories before and after continuing education when analytic sample is restricted to participants without evidence of cognitive impairment (N=1,790).

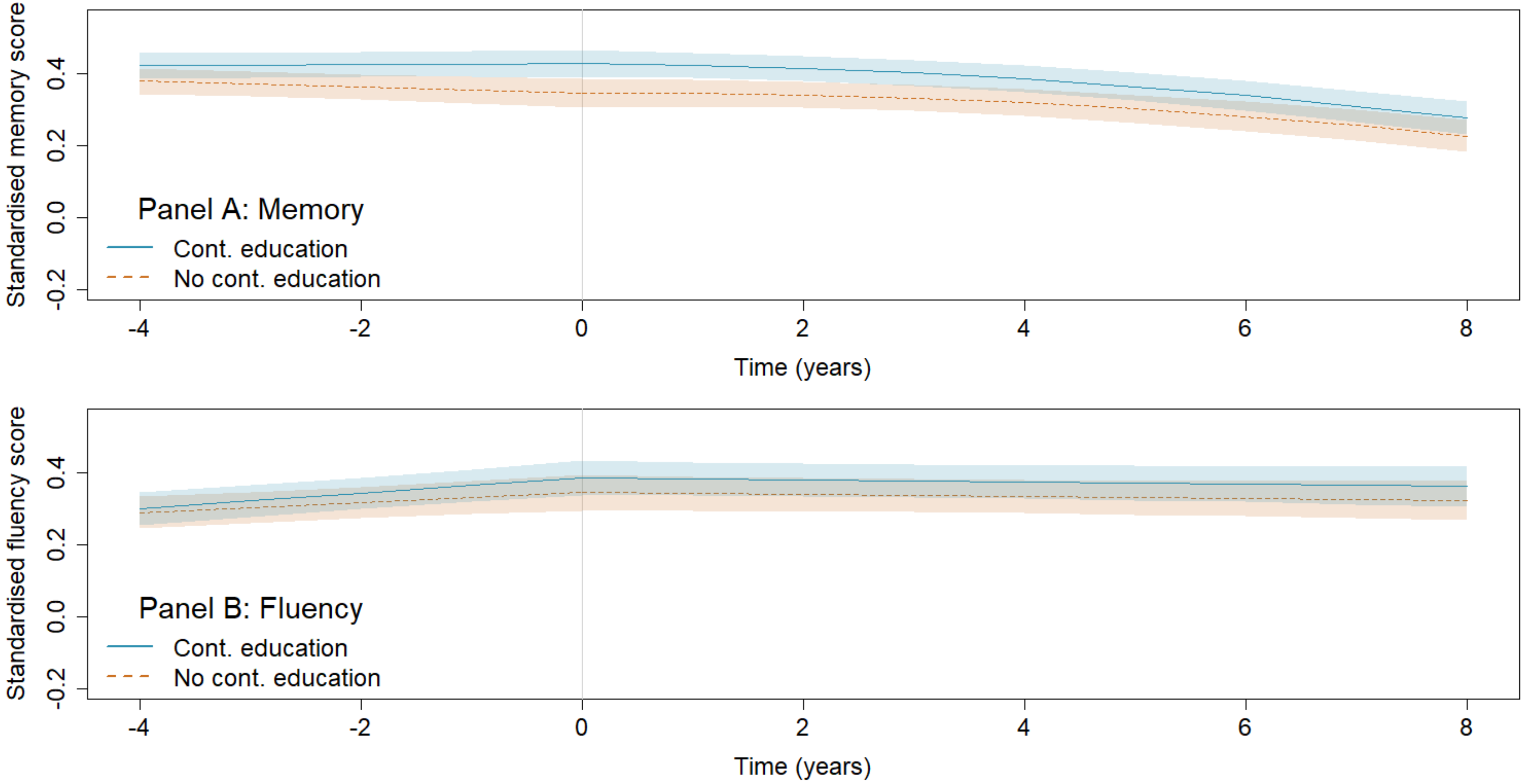

Predicted cognitive scores averaged over the observed covariate distribution in the analytic sample, shown over the median follow-up periods of four years before and eight years after continuing education (time = 0) or the equivalent time interval in the control group. Analyses are restricted to those without cognitive scores suggesting cognitive impairment or reporting a dementia diagnosis (Methods S3). Models were adjusted for age at time = 0, sex, baseline education level, labour force status, wealth, health behaviours, self-rated hearing, depressive symptoms, and long-term conditions.

Figure S9. Average cognitive trajectories before and after continuing education when analytic sample is restricted to participants reporting continuing education in the last month (N=1,478).

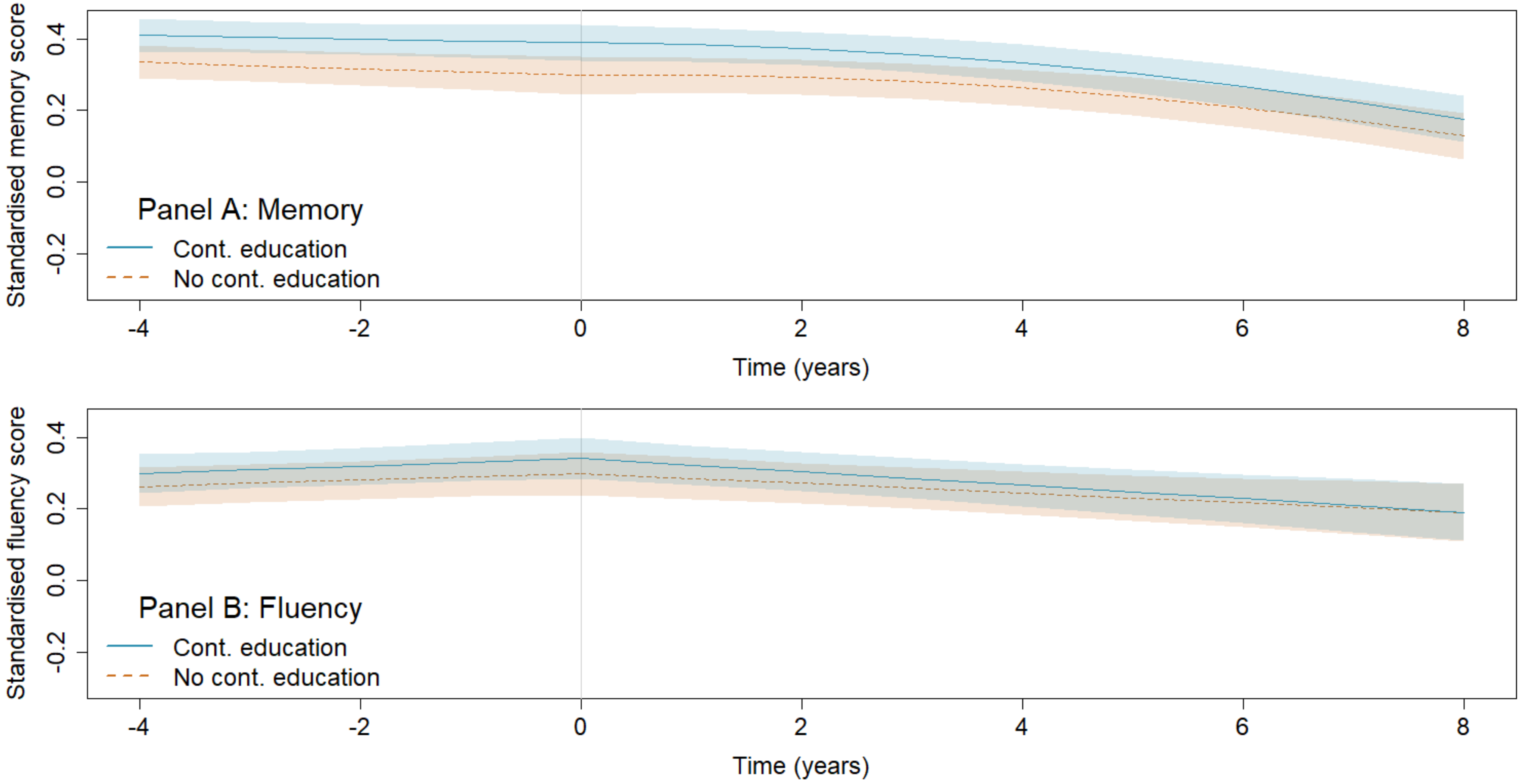

Predicted cognitive scores averaged over the observed covariate distribution in the analytic sample, shown over the median follow-up periods of four years before and eight years after continuing education (time = 0) or the equivalent time interval in the control group. Analyses were restricted to those reporting continuing education within the last month and their controls. Models were adjusted for age at time = 0, sex, baseline education level, labour force status, wealth, health behaviours, self-rated hearing, depressive symptoms, and long-term conditions.

4. References.

1. Turvey CL, Wallace RB, Herzog R. A Revised CES-D Measure of Depressive Symptoms and a DSM-Based Measure of Major Depressive Episodes in the Elderly. *International Psychogeriatrics* 1999; **11**(2): 139-48.
2. Blackwell M, Iacus S, King G, Porro G. cem: Coarsened exact matching in Stata. *The Stata Journal* 2009; **9**(4): 524-46.
3. Iacus SM, King G, Porro G. Causal inference without balance checking: Coarsened exact matching. *Political analysis* 2012; **20**(1): 1-24.
4. Scott DW. Sturges' rule. *Wiley Interdisciplinary Reviews: Computational Statistics* 2009; **1**(3): 303-6.
5. Young M. National vocational qualifications in the United Kingdom: their origins and legacy. *Journal of Education and Work* 2011; **24**(3-4): 259-82.
6. Chertkow H, Nasreddine Z, Joanette Y, et al. Mild cognitive impairment and cognitive impairment, no dementia: Part A, concept and diagnosis. *Alzheimers Dement* 2007; **3**(4): 266-82.
